# Psoriasis as risk factor for non-ischemic dilated cardiomyopathy: a population-based cross-sectional study

**DOI:** 10.1186/s12872-021-01972-0

**Published:** 2021-03-31

**Authors:** Abbas Alshami, Nasam Alfraji, Steven Douedi, Swapnil Patel, Mohammad Hossain, Deborah Alpert, Dawn Calderon

**Affiliations:** 1 Department of Medicine, Jersey Shore University Medical Center, 1945 Route 33, Neptune, NJ 07753 USA; 2 Department of Rheumatology, Jersey Shore University Medical Center, Neptune, NJ USA; 3 Department of Cardiology, Jersey Shore University Medical Center, Neptune, NJ USA

**Keywords:** Psoriasis, Psoriatic arthritis, Non-ischemic dilated cardiomyopathy, Cardiovascular disease, Autoimmune disease

## Abstract

**Background:**

Psoriasis is a chronic inflammatory skin condition commonly associated with psoriatic arthritis, malignancy, diabetes, inflammatory bowel disease, and cardiovascular disease. Several reports and studies have reported an association between psoriasis and non-ischemic dilated cardiomyopathy (NIDCM). We aim to study the relationship between psoriasis and non-ischemic dilated cardiomyopathy in a large population-based study.

**Methods:**

We utilized the Healthcare Cost and Utilization Project National Inpatient Sample 2017 database, which represents a 20% sample of all payer hospitalizations in the United States. We investigated hospitalizations for patients aged 18 years old or older with diagnoses of any type of psoriasis and non-ischemic dilated cardiomyopathy. Psoriasis, cardiomyopathy, and other comorbidities were identified through their international classification of diseases, 10th revision codes recorded in the discharge record for each hospitalization.

**Results:**

Of a total of 6,084,184 all-cause admissions, 0.5% were admissions for patients with psoriasis (n = 32,807). Of the patients with and without psoriasis who had non-ischemic dilated cardiomyopathy, after adjusting for age, sex, race, diabetes mellitus, hypertension, alcohol abuse, cocaine abuse, arrhythmias, and obesity in a multivariate analysis, the presence of psoriasis was not significantly associated with non-ischemic dilated cardiomyopathy.

**Conclusion:**

Psoriasis is a chronic autoimmune disorder which carries a higher cardiovascular events and more prevalent traditional atherosclerotic risk factors in comparison to the general population. However, association with non-ischemic cardiomyopathy or NIDCM in particular has not been studied sufficiently. Our study, being one of the first larger studies to assess this correlation, indicated no relationship between psoriasis and non-ischemic dilated cardiomyopathy.

## Introduction

Psoriasis is a chronic inflammatory skin condition which commonly presents with scaly erythematous plaques. It has a worldwide prevalence ranging from 0.5 to 11% [1]. Psoriasis is an immune mediated disease, with T-Cells, dendritic cells, and cytokines playing a crucial role in skin damage [2]. The most common complication or association with psoriasis is psoriatic arthritis, which involves the musculoskeletal system [3]. Other conditions include but are not limited to malignancy, diabetes, inflammatory bowel disease, and several studies have implied a possible relationship between psoriasis and cardiovascular disease [4–13]. While the relationship between psoriasis and cardiomyopathies, especially non-ischemic dilated cardiomyopathy (NIDCM), has been suggested through case reports and small observational studies, the association has not been evaluated using a large sample. This population-based study aims to investigate the correlation between patients with psoriasis and non-ischemic dilated cardiomyopathy.

## Methods

### Study design/settings

We conducted a cross sectional study for adults hospitalized for any cause at any hospital in 2017 in the United States. We utilized the Healthcare Cost and Utilization Project National Inpatient Sample (HCUP-NIS) 2017 database, which represents a 20% sample of all payer hospitalizations in the United States. These hospitalizations were selected systematically by the Agency for Healthcare Resources and Quality (AHRQ), thus represent all the hospitalizations in the United States in 2017. Notably, these encounters represent hospitalizations, not patients, so multiple potential hospitalizations for the same patient are considered as multiple encounters.

### Participants

We included all hospitalizations for patients ≥ 18 years old. We divided them to two main groups, admissions with and without psoriasis. Then, we identified admissions with and without NIDCM, as well as other clinical entities, in each main group. Encounters for patients aged < 18 years old were excluded due to differences in risk factors and pathophysiology of cardiomyopathy between the pediatric and adult populations.

### Variables

The data collected in the database included demographic variables, admission diagnoses, procedures, type of insurance, geographical location, length of stay, and inpatient mortality, among others. Every encounter can include up to 40 diagnoses and are coded using the international classification of diseases, 10th revision (ICD-10 codes).

### Outcomes

We aimed to investigate if psoriasis represents an independent risk factor for NIDCM.

### Data measurement

Admission diagnoses, including psoriasis, NIDCM, and other comorbidities and substances were identified through their ICD-10 codes recorded in the discharge record for each hospitalization. “Appendix” contains a list of the ICD-10s used in this study.

### Ethical considerations

Institutional review board (IRB) is not required to for studies utilizing the national inpatient sample, as it falls under the “limited data sets” category exempted by the HIPAA privacy regulations (http://privacyruleandresearch.nih.gov). The study was conducted in agreement with the principles of the Declaration of Helsinki.

### Statistical methods

Continuous and categorical variables were described as mean with standard deviation (SD) and frequencies, as appropriate. Chi square and t-test were used to compare categorical and continuous variables, as appropriate. Multivariate analyses were performed to determine if psoriasis represents an independent risk factor for cardiomyopathies. All analyses were done using IBM SPSS Statistics™ version 25.0 (IBM Corporation, Artmonk, NY). An alpha value (*p*) of 0.05 was used to ascertain statistical significance.

## Results

Of a total of 6,084,184 all-cause admissions, 0.5% were admissions for patients with psoriasis (n = 32,807).

### Baseline characteristics

Baseline characteristics differed between admissions with and without psoriasis and are summarized in Table 1. Mean age was 61 years with SD of 15.4 years for patients with psoriasis, as opposed to mean age of 57 years with SD of 20.2 years in admission for patients without psoriasis. Around half of the encounters were for female patients, and White was the most prevalent race for both populations. Generally, comorbidities were more prevalent in patients with than without psoriasis.

**Table 1:** Baseline characteristics of all admitted patients and patients with psoriasis

	All admissions	Admissions with psoriasis	*p* value
	Mean (SD) or N (%)	Mean (SD) or N (%)	
Age (years)	57.9 (20.3)	61.2 (15.4)	< 0.001
*Sex*			< 0.001
Male	2,554,599 (42.2)	16,388 (50)	
Female	3,496,439 (57.8)	16,416 (50)	
*Race*			< 0.001
White	3,924,190 (67.2)	26,032 (79.3)	
Black	891,986 (15.3)	1782 (5.4)	
Hispanic	649,121 (11.1)	2275 (6.9)	
Asian or Pacific Islander	161,026 (2.8)	668 (2)	
Native American	361,77 (0.6)	195 (0.6)	
Other	178,555 (3.1)	755 (2.3)	
Hypertension	3,342,609 (55.2)	21,505 (65.6)	< 0.001
Diabetes Mellitus	1,623,171 (26.8)	10,831 (33)	< 0.001
Arrhythmias	999,060 (16.5)	5706 (17.4)	< 0.001
Hyperthyroidism	30,933 (0.5)	183 (0.6)	0.238
Smoking	981,827 (16.2)	6195 (18.9)	< 0.001
Obesity	955,616 (15.8)	8639 (26.3)	< 0.001
Alcohol abuse	1,153,177 (19.1)	10,241 (31.2)	< 0.001
Cocaine abuse	84,584 (1.4)	347 (1.1)	< 0.001
Long term use of corticosteroids	93,308 (1.5)	1283 (3.9)	< 0.001
History of chemotherapy/immunotherapy	85,623 (1.4)	480 (1.5)	0.461
History of radiotherapy	81,775 (1.4)	493 (1.5)	0.02
Sarcoidosis	16,254 (0.3)	158 (0.5)	< 0.001
Total	6,084,184 (100)	32,807 (100)	

### Psoriasis and NIDCM

Of all-cause admission records, 0.7% had NIDCM, as opposed to 0.9% in patients with psoriasis (*p* = 0.03). However, after adjusting for all the variables listed in Table 1 in a multivariate analysis, psoriasis failed to survive as an independent predictor of NIDCM (Table 2).

**Table 2:** Multivariable logistic regression model to predict non-ischemic cardiomyopathies

Variable	OR	95% CI for OR	*p* value
Age	0.989	0.988–0.990	< 0.001
Sex, female	0.472	0.462–0.482	< 0.001
Race, White	1		Ref
Race, Black	2.280	2.228–2.333	< 0.001
Race, Hispanic	1.271	1.228–1.315	< 0.001
Race, Asian or Pacific Islander	1.352	1.270–1.439	< 0.001
Race, Native American	1.711	1.536–1.906	< 0.001
Race, other	1.079	1.013–1.150	0.018
Diabetes Mellitus	1.035	1.014–1.056	0.001
Hypertension	2.615	2.541–2.690	< 0.001
Alcoholism	1.639	1.593–1.688	< 0.001
Smoking	1.175	1.146–1.205	< 0.001
Cocaine use	1.429	1.347–1.516	< 0.001
Arrhythmias	5.191	5.082–5.302	< 0.001
Obesity	0.819	0.793–0.845	< 0.001
Sarcoidosis	1.541	1.365–1.741	< 0.001
Psoriasis	1.030	0.912–1.165	0.632
History of chemo/immunotherapy	1.428	1.320–1.544	< 0.001
History of radiotherapy	0.964	0.886–1.049	0.391
Hyperthyroidism	1.498	1.362–1.648	< 0.001

When NIDCM was further subdivided into different causes, only viral and alcoholic cardiomyopathies were statistically associated with psoriasis in bivariate analyses (Chi-square test) (Table 3). Multivariable analyses using multivariable logistic regression models showed that psoriasis survived as an independent risk factor for viral CM with marginal statistical significance (adjusted OR 2.3, 95% CI 1.020–5.131, *p* = 0.045), while it failed to predict alcoholic CM (adjusted OR 0.941, 95% CI 0.651–1.359, *p* = 0.74).

**Table 3:** Bivariate correlations between psoriasis and different types of cardiomyopathies

Cardiomyopathy type	Frequency	N with psoriasis (%)	*p* value
Idiopathic	39,943	239 (0.6)	0.11
Peripartum CM	903	1 (0)	0.08
Alcoholic CM	4105	36 (0.9)	0.003
Drug induced CM	2626	11 (0.4)	0.40
Viral CM	456	6 (1.3)	0.02
Total NICM	46,369	285 (0.6)	0.03

## Discussion

This is a cross sectional study aimed to investigate the association between psoriasis and NIDCM. We found that NIDCM was more prevalent in patients with psoriasis; however, this association is likely due to higher prevalence of risk factors of NIDCM, rather than psoriasis. The only exception was viral CM, which was 2.3 times more likely to affect patients with psoriasis. We also found that arrythmias, followed by hypertension, black race, and male gender were the strongest predictors of NIDCM. Other independent predictors included native American race, alcoholism, sarcoidosis, hyperthyroidism, and cocaine use, among others (Table 2).

Several studies have described the higher prevalence of cardiovascular diseases such as acute coronary disease and cerebrovascular events in psoriatic patients comparing to the general population [5–13]. However, the prevalence of cardiomyopathy in those patients has not been well studied. The prevalence of psoriasis in our study was 0.5%, with mean age of our psoriatic patients was 61 years (± 15.4) years comparing to a mean age of 57 (± 20.2) years for all cause admission. Regarding ethnicity, Caucasians were more prevalent in our data in both populations. However, psoriasis has been noted to be more prevalent in white race historically [14]. We found an equal distribution between males and females.

Despite several previous studies and data that demonstrated the association of adverse cardiovascular risk factors and events with psoriasis or psoriatic arthritis [5–13], our present study found that no increased occurrence of NIDCM with any type of psoriasis after adjusting for the traditional risk factors such as age, sex, race, diabetes mellitus, hypertension, alcohol abuse, cocaine abuse, arrythmias, and obesity. Therefore, psoriasis cannot be considered as independent risk factor for NIDCM according to our study.

Nonetheless, upon literature review, an interesting relationship between psoriasis and non-ischemic cardiomyopathy has been increasingly reported in few case reports and studies. Most of these cases were associated with non-ischemic dilated cardiomyopathy; however, few reports described hypertrophic cardiomyopathy in psoriasis patients [15–18]. Eliakim-Raz et al. investigated 2292 psoriasis patients in 2008 for cardiomyopathy in one large hospital in Israel. This study reported cardiomyopathy of different types in 20 of these psoriasis patients (a prevalence of 0.87%). Ten of the twenty were diagnosed with dilated CM (a prevalence of 0.43% among psoriasis patients) and most of them had normal coronaries [15]. A case report by Abdelaoui et al. also revealed NIDCM in a patient with a long history of psoriasis [16]. Pietrzak et al. reported dilated cardiomyopathy with a severe ventricular impairment higher than it could result from myocardial ischemia in psoriasis patient [17]. Moreover, Fukuhara et al. reported an association between psoriatic arthritis, Takayasu’s disease, and non-ischemic dilated CM, which supposedly suggests a common underlying immune-mediated inflammatory process among the three conditions [18].

Another cohort study by Zhao et al. demonstrated that patients with severe psoriasis showed subclinical myocardial (left ventricular systolic) dysfunction as detected by 2D speckle tracking derived strain analysis compared with control group [19]. Milaniuk et al. screened the echocardiography of patients with psoriasis and psoriatic arthritis and reported left ventricle diastolic dysfunction in 27.8% of the patients, and left ventricle hypertrophy in 11.1%. Left ventricular systolic dysfunction has been noticed as well [20]. Similarly, a large study conducted by Biyik et al. in one of the hospitals in Turkey in 2006 found that the longer is the disease, the higher incidence of left ventricular diastolic dysfunction is in psoriasis patients [21].

Studies have proposed few possible hypotheses that would lead to NIDCM in psoriasis patients [22, 23]. One of them suggests that psoriasis-induced myocardial inflammation will eventually results in a form of inflammatory non-ischemic dilated cardiomyopathy involving certain growth factors and cytokines produced by inflammatory cells such as transforming growth factor-β, interleukin-1 (IL-1), and interleukin-17A (IL-17A) [22]. Another article by Ryabkova et al. illustrated an interesting autoantibody-mediated mechanism engaging several classes of autoantibodies (AAb) such as cytotoxic autoantibodies or cell-modulating autoantibodies. Cytotoxic antibodies, such as anti-cardiac troponin I Ab, anti-α- and anti-β-myosin heavy chain Ab and anti-mitochondrial Ab, can cause antibody-dependent cardiomyocyte death, accompanied by severe inflammation with increase in the local concentration of tumor necrosis factor (TNF-α), nitric oxide, and cytokines. On the other hand, cell-modulating autoantibodies have dysregulating effects resulting in alteration of cell function, growth and signaling and eventually leading to contractility and/or conduction disorders [23].

Interestingly, on bivariate analysis of the psoriasis patients in our study, the prevalence of alcoholic and viral cardiomyopathies was higher in this population compared to the general population, at 0.9% and 1.3%, respectively. Having said that, after adjusting for potential confounding factors in multivariable regression models, only viral CM seemed to maintain the statistical significance (OR 2.3, 95% CI 1.020–5.131, *p* = 0.045). The higher prevalence of alcoholic CM in patients with psoriasis can be attributed to the higher prevalence of alcoholism in patients with psoriasis (31% vs 19%, Table 1). Although not statistically significant, the association between alcoholic CM and psoriasis is noticeably observed at 0.941% in our study which could be attributed to the psychosocial aspects and consequences of this debilitating autoimmune disease [24, 25]. As viral cardiomyopathy is the only type that has been noticed to be associated with psoriasis in our current study, several explanations could justify this higher incidence. One of the reasons is that psoriasis patients have immune dysfunction that could put them at a higher risk for infections and its complications, including viral infections, compared to the general population [26].

Our study is one of the first studies to highlight the association between NIDCM and any type of psoriasis; therefore, we cannot disclose a definitive conclusion, especially given the cross-sectional design of the study that is insufficient to establish causality. More robust and larger studies could illustrate the causality between viral and alcoholic cardiomyopathies and psoriasis. In addition, including inflammatory markers in future studies can provide additional information about the existence of such association.

We observe that our study has several limitations that might impact the application of its findings and must be acknowledged. First, severity of the disease in psoriatic patients was not available, therefore the association between severity of psoriasis and cardiomyopathy cannot be assessed. Second, HCUP-NIS database does not provide information on other important confounders such as anti-psoriatic immunosuppressive medications that could cause cardiomyopathy as a side effect. Finally, our study is cross sectional which analyzes the prevalence at single point time and subsequently cannot assess the influence of the disease duration on precipitating cardiomyopathy.

## Conclusion

Psoriasis is a chronic autoimmune disorder which traditionally carries a high cardiovascular risk including accelerated atherosclerosis and ischemic CM in comparison to the general population. Moreover, previous data has suggested that aggressive treatment of the disease as well as better control of the traditional atherosclerotic risk factors may reduce cardiovascular events in psoriatic patients. Despite the interesting relation between psoriasis and NIDCM that has been reported increasingly in few case reports and studies, our current study indicated no correlation between psoriasis and NIDMC, except viral type, although we cannot reach a definitive conclusion from a single study and the aforementioned limitations might have influenced the results. Therefore, further research especially prospective studies are recommended to illuminate the exact correlation between all different subclasses of psoriasis and non-ischemic dilated cardiomyopathy.

## Data Availability

The datasets generated and/or analyzed during the current study are available in the Healthcare Cost and Utilization Project National Inpatient Sample (HCUP-NIS) 2017 database repository (https://www.hcup-us.ahrg.gov/nisoverview.jsp).

## Appendix

List of ICD-10 used in the study:

1. Psoriasis: “L40” and its subclassifications.
2. Peripartum cardiomyopathy: “O90.3”.
3. Viral cardiomyopathy: “B33.24”.
4. Idiopathic dilated cardiomyopathy: “I42.0”
5. Alcoholic cardiomyopathy: “I42.6”
6. Drug induced cardiomyopathy: “I42.7”
7. Non-ischemic cardiomyopathies: ICD-10 of points 2–6 above.
8. Diabetes mellitus: “E08”, “E09”, “E10”, “E11”, “E12”, “E13” and their subclassifications.
9. Hypertension: “I10”, “I11”, “I12”, “I13”, “I15” and their subclassifications.
10. Arrhythmias: “I47”, “I48” and their subclassifications.
11. Alcohol: “F10” and its subclassification.
12. Cocaine: “F14” and its subclassification.
13. Obesity: “E66”, “Z68.3”, “Z68.4” and subclassifications.
14. Smoking: “F17” and subclassifications.
15. Sarcoidosis: “D86” and subclassifications.
16. Hyperthyroidism: “E05” and subclassifications.
17. Chemo/Immunotherapy: “Z92.25”, “Z92.21”.
18. Radiotherapy: “Z92.3”.

## Abbreviations

NIDCM: Non-ischemic dilated cardiomyopathy
IRB: Institutional review board
CM: Cardiomyopathy
