# Correction to: Psoriasis as risk factor for non-ischemic dilated cardiomyopathy: a population-based cross-sectional study

**DOI:** 10.1186/s12872-021-01984-w

**Published:** 2021-04-14

**Authors:** Abbas Alshami, Nasam Alfraji, Steven Douedi, Swapnil Patel, Mohammad Hossain, Deborah Alpert, Dawn Calderon

**Affiliations:** 1 Department of Medicine, Jersey Shore University Medical Center, 1945 Route 33, Neptune, NJ 07753 USA; 2 Department of Rheumatology, Jersey Shore University Medical Center, Neptune, NJ USA; 3 Department of Cardiology, Jersey Shore University Medical Center, Neptune, NJ USA

## Correction to: Alshami et al. BMC Cardiovasc Disord (2021) 21:161 https://doi.org/10.1186/s12872-021-01972-0

Following the publication of the original article [1], an error was identified in the Acknowledgements section.

The sentence “The authors would like to acknowledge all the 174 collaborators who facilitated data collection in their institutions” should be replaced with “Not applicable”.

The original article has been corrected as well.
